# High diversity stabilizes the thermal resilience of pollinator communities in intensively managed grasslands

**DOI:** 10.1038/ncomms8989

**Published:** 2015-08-10

**Authors:** Sara Kühsel, Nico Blüthgen

**Affiliations:** 1 Department of Biology, Technische Universität Darmstadt, Schnittspahnstr. 3, D-64287, Darmstadt, Germany

## Abstract

The resilience of ecosystems depends on the diversity of species and their specific responses to environmental variation. Here we show that the diversity of climatic responses across species contributes to a higher projected resilience of species-rich pollinator communities in real-world ecosystems despite land-use intensification. We determined the thermal niche of 511 pollinator species (flies, bees, beetles and butterflies) in 40 grasslands. Species in intensively used grasslands have broader thermal niches and are also more complementary in their thermal optima. The observed increase in thermal resilience with land-use intensification is mainly driven by the dominant flies that prefer cooler temperatures and compensate for losses of other taxa. Temperature explained 84% of the variation in pollinator activity across species and sites. Given the key role of temperature, quantifying the diversity of thermal responses within functional groups is a promising approach to assess the vulnerability of ecosystems to land-use intensification and climate change.

## Abstract (web summary)

How variation in species responses to climate may influence the resilience of ecological communities to environmental change is not fully understood. Here, the authors characterize the thermal niches of insect pollinator communities and show that resilience increases along a gradient of land-use intensity.

## Graphical abstract

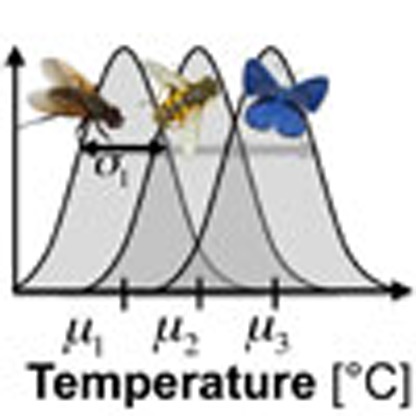

Ecosystem resilience is a pivotal concept in different contexts ranging from the production of natural resources and other ecosystem services to the conservation of species and natural systems^1^,^2^,^3^. Species diversity within a functional group (functional redundancy) often stabilizes ecosystem functioning by providing insurance against losses of single species (insurance hypothesis), or by dampening individual species fluctuations (portfolio effect)^4^,^5^. In simple words, when a species is lost, functionally redundant (or ‘equivalent') species may replace its performance temporarily or in the long term. Indeed, for a number of ecosystem services, positive relationships between diversity and stability have been confirmed^6^. In theory, stability arises from the extent to which ‘redundant' species differ in multiple other niche dimensions^7^, particularly their responses to environmental conditions^8^,^9^. Such a response diversity^1^ of functionally redundant species ensures a higher probability that at least some species continue to perform their functions—stabilizing an ecosystem over time when conditions vary^8^,^9^,^10^,^11^.

Whereas the stabilizing role of response diversity is intriguing theoretically, quantitative models are scant, focal environmental conditions are rarely specified and empirical data from real ecosystems are largely missing^12^. Instead of using morphological or life-history traits^13^, response diversity may also be defined for target environmental variables. For instance, Cariveau *et al.*^14^ assessed the linear abundance—land use relationship for different species of bees and then defined the variation of slopes across co-occurring species as a measure of response diversity. Their response diversity did not translate into a consistent stabilization of crop pollination services. However, in such concepts based on monotonous or linear environmental responses, stability may not necessarily increase with the diversity of slopes of land-use responses, but by positive slopes *per se* (that is, species' tolerances of land use).

In addition to land-use responses^14^ or vulnerability to disturbance^12^ we can also quantify response diversity based on well-defined environmental niche dimensions such as climatic conditions^8^,^9^. In the present study, we focus on thermal niches of pollinators to characterize response diversity and resilience. Responses to temperature are relevant for the behaviour, phenology and distributional ranges for animals, particularly ectotherms and their ecosystem functioning such as pollination^15^,^16^. Thermal niches are typically uni-modal^17^,^18^. Species differ in their thermal tolerance (niche breadth) as well as in their optima (niche complementarity) (Fig. 1). The ‘community niche' is composed of individual species niches that co-occur at the same site. Here we explicitly define the integral defined by the community niche of all co-occurring species that perform a particular function—pollinators—as a proxy of the functional resilience of an ecosystem. Ecological resilience is traditionally defined as the ability of an ecosystem to absorb environmental changes^19^,^20^; here specifically the ability to maintain a functional performance level, such as pollinator visitation against variation in climatic conditions. Consequently, broader tolerances of individual species' performances (niche breadth) and the extent of variation across species (niche complementarity) both contribute positively to functional resilience. Fig. 1 visualizes our general concept and definition of response diversity and resilience for thermal responses that can also be generalized to other environmental variables and uni-modal reaction norms^8^,^9^. Whereas resilience in a strict sense involves measuring thresholds for ecosystem transitions that are rarely applicable to real communities^20^, our mathematical framework provides a practicable, explicit quantitative prediction for resilience that goes beyond a mere characterization of ‘functional diversity'.

Different processes are crucial for the maintenance of ecosystems, among which the biomass productivity of plants is the best understood ecosystem function in the context of biodiversity experiments^6^,^21^. Pollination by animals is important for three-quarters of the major crop plants^22^ and for the reproduction of an even higher proportion of wild plants^23^. Numerous taxa visit flowers and are potential pollinators^24^ such as bees, flies, butterflies and beetles. Nevertheless there are concerns regarding the maintenance of pollination services because of recent large-scale declines in pollinator diversity^25^,^26^, which is largely caused by intensive land use^22^,^27^,^28^. High fertilizer application and frequent mowing or grazing may lead to impoverished grasslands with structurally homogenous rewards^29^. Grazing and cutting remove floral food recourses and accordingly affect pollinators^30^. However, in the same grasslands as examined in the present study, Weiner *et al.*^31^ found that total pollinator diversity generally remained at a high level, irrespective of land-use intensity (LUI). Whereas diversity was constant, the composition of the pollinator community changed considerably. In intensively used grasslands flies became more dominant whereas butterflies and bees were less frequent. A higher dominance of a taxon, a case of homogenization, likely correspond to more similar response traits and could lead to decreasing response diversity such as a smaller thermal activity range of pollinators.

Little is known about the variance in climatic responses between species in a community, and how this interspecific variation represents a stabilizing element to fluctuating conditions. We aim to extrapolate variation in thermal niches between species to the community level, and to study whether the diversity of thermal responses and the projected thermal resilience of pollinator communities are affected by LUI. First, we quantified the importance of temperature in predicting the general activity of pollinators. Second, we studied the changes in thermal niches of pollinator communities with LUI and whether the projected resilience corresponds to pollinator diversity. Third, we examined how thermal niches vary across different pollinator taxa and with body size. To answer these questions, we investigated pollinator communities on 40 experimental grassland sites along a land-use gradient during different daily and seasonal temperature conditions. Our results show that thermal resilience of pollinator communities increases with LUI, an effect mainly driven by flies that become more dominant. Some species thus maintain ecosystem functions at unfavourable conditions—a consequence of the high level of diversity that occurs along the land-use gradient.

## Results

### General findings

In total 14,873 pollinator individuals from 511 species and 64 families belonging to the orders Diptera (64%), Hymenoptera (28%), Coleoptera (5%) and Lepidoptera (3%) were collected from 40 plots. A total of 143 species of plants were flowering during our surveys, of which 104 were visited by pollinators. The species diversity of both flowering plants and pollinators was high: the mean effective diversity (*e*^*H'*^) per plot per day was 4.43±1.82 for flowers and 26.05±10.86 for pollinators. LUI had a negative impact on flower diversity (linear model (lm), LUI: F_1,36_=5.5, *P*=0.024, region: *F*_1,36_=4.5, *P*=0.041; significant in the Schwäbische Alb but not in the Hainich). In contrast, pollinator diversity did not change with LUI (lm, LUI: *F*_1,36_=0.1, *P*=0.81, region: *F*_1,36_=3.8, *P*=0.056).

**Figure 1:**
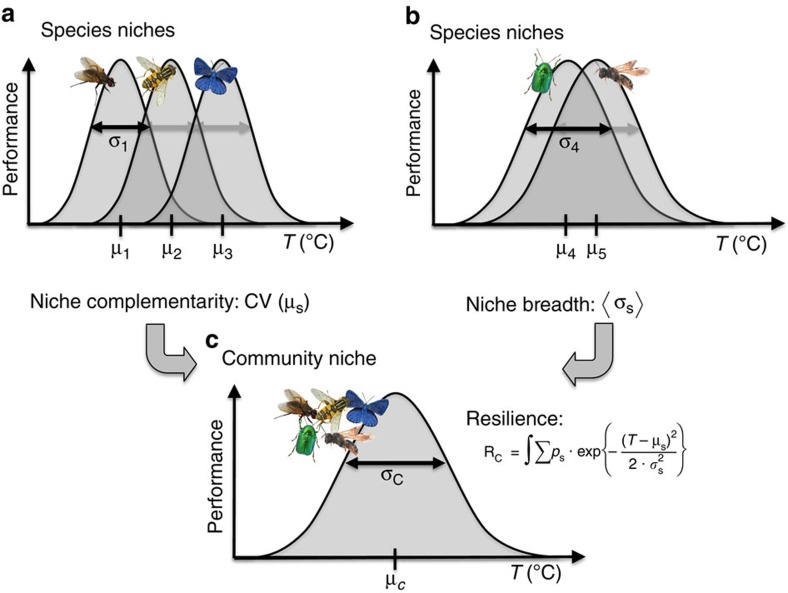
Conceptual framework of community (thermal) niche, response diversity and resilience. A broader community niche and higher thermal resilience may be driven by (**a**) high niche complementarity of species, that is, strong variation in species mean niche positions (μ_S_) and thus response diversity, and/or by (**b**) broader species niches (*σ*_S_). The community resilience (*R*_C_) is based on the weighted sum of the individual species niches (**c**), with species weighted by their proportional abundance (*p*_S_).

The composition of the plant community changed significantly with LUI. The proportion of *Asteraceae* on total flower cover per plot increased from about 10% on extensive plots to over 50% on intensively used plots, whereas the proportion of *Fabaceae* decreased from 40 to 10%. The proportion of other abundant plant families did not change significantly (see Supplementary Table 1, Fig. 1). We also found changes in the composition of the pollinator community with LUI. The proportion of flies increased significantly with LUI (lm, *F*_1,38_=5.1, *P*=0.029) except hoverflies that showed the opposite trend (*F*_1,37_=3.3, *P*=0.079). The proportion of butterflies decreased with LUI (*F*_1,26_=9.9, *P*=0.004), as well as bees in the Schwäbische Alb (*F*_1,14_=13.3, *P*=0.003), but not in the Hainich (*F*_1,22_=0.2, *P*=0.698). There was no consistent change in the proportion of other hymenopterans and beetles.

### Thermal niche

**Figure 2:**
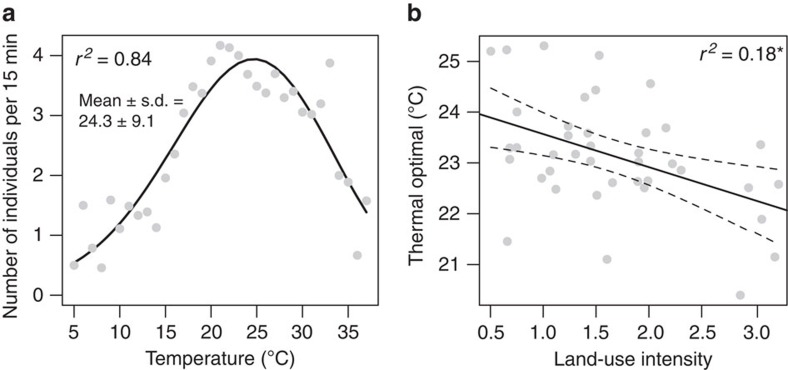
Temperature preferences of pollinators. Effects of temperature on total pollinator activity (**a**), where each data point corresponds to the mean number of individuals recorded per 15 min during a given temperature (all surveys in 2012 pooled). (**b**) Community thermal optima (weighted means across all species occurring in a plot) in response to land-use intensity. Significances: *P<0.05, **P<0.01, ***P<0.001. Dotted lines represent 95% confidence intervals.

The activity of pollinators was highly correlated with air temperature, as expected for ectotherms. Some 84% of the variation in total pollinator activity was explained by the temperature, closely following a Gaussian function (Fig. 2a). In contrast, flower cover on the plots during the surveys had no consistent effect on pollinator abundance (lm, *F*_1,37_=2.4, *P*=0.13), confirming the primary importance of temperature for pollinator activities. To investigate differences in the thermal niches of pollinators, we calculated for all 511 species a thermal optimum (μ_S_) and for 406 species (all with *N*>1) a thermal niche breadth (*σ*_S_). The mean thermal optimum of a community (μ_C_) decreased with LUI (Table 1, Fig. 2b), indicating that pollinator species in intensively used grasslands preferred colder temperatures than those on extensive grasslands. Thermal optima μ_S_ varied across pollinator taxa (Fig. 4b). Bees and butterflies preferred warmer temperatures than other hymenopterans, flies and beetles.

**Figure 3:**
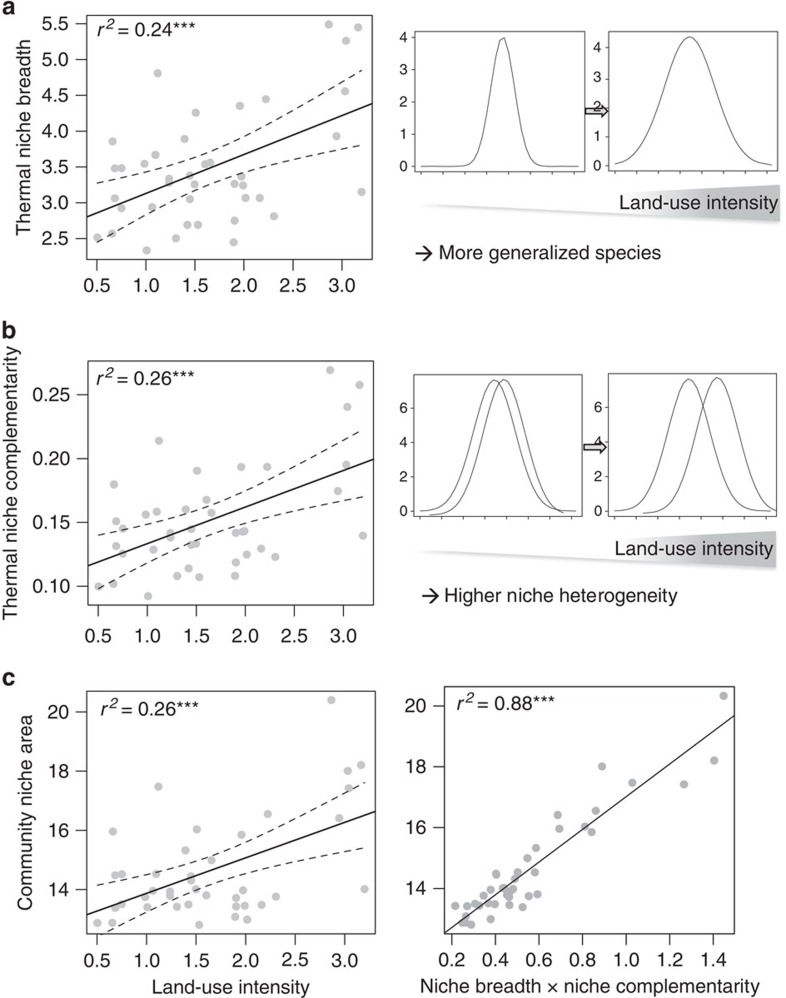
Thermal niches of pollinators depending on land-use intensity. Effects of land-use intensity on thermal niche breadth (**a**), niche complementarity (**b**) and community niche area (**c**). Community niches were predicted by the product of niche breadth and complementarity (**d**). Dotted lines represent 95% confidence intervals.

Average species niche breadths <*σ*_S_> (Fig. 3a, Table 1) as well as niche complementarity CV_C_ (Fig. 3b, Table 1) significantly increased with LUI. Thermal generalists were thus more common in intensively used grasslands, and co-existing species in such grasslands differed to a greater extent in their thermal optima. Both effects were independent of pollinator diversity (Table 1). In consequence, community niche area (*R*_C_) as an appropriate proxy measure for resilience, increased with LUI (Fig. 3c), and the effect was consistent in both regions (Table 1). The product of <*σ*_S_> and CV_*C*_ strongly predicted the variation in *R*_*C*_ (lm, *F*_1,38_=16.8, *P*<0.001, Fig. 3c). The resilience *R*_*C*_ considers variability along the entire temperature range (5–40 °C), thus we additionally examined how these communities may respond to warmer conditions (35–40 °C) that become increasingly common with global warming. Despite the negative trend in μ_S_, a high level of resilience *R*_*C*_ for warm conditions was maintained across the LUI gradient (linear mixed model (lme), *F*_1,35_=3.8, *P*=0.060; with a marginally significant increase that was consistent across regions, *F*_1,35_=0.5, *P*=0.181). For pollinator data from 2008, recorded on 70 grassland plots in the same regions, we confirmed a marginally significant trend that niche breadth, complementarity and community niche area increased with LUI (see Supplementary Table 2).

Pollinator taxa differed in their thermal niches breadth and niche complementarity. Butterflies, hoverflies and bees had narrower niches and also a lower niche complementarity across the species than other flies, other hymenopterans and beetles (Table 2). Note that variation in niches is independent of number of species in each taxon, for example, other hymenopterans contain relatively few species that were highly complementary whereas other flies or bees contained more species that were more similar in their thermal niches. Niche breadth und complementarity were even more variable at family level (see Supplementary Fig. 2).

Considering that flies became more dominant on intensively used plots we investigated whether effects of LUI on the thermal responses of the community were mainly driven by flies or by other taxa. Niche breadth, complementarity and community niche area of flies alone were positively related to LUI (Table 1), consistent with the trend found for all taxa. For the remaining taxa pooled, we found no effect of LUI on thermal responses (see Supplementary Table 3).

**Figure 4:**
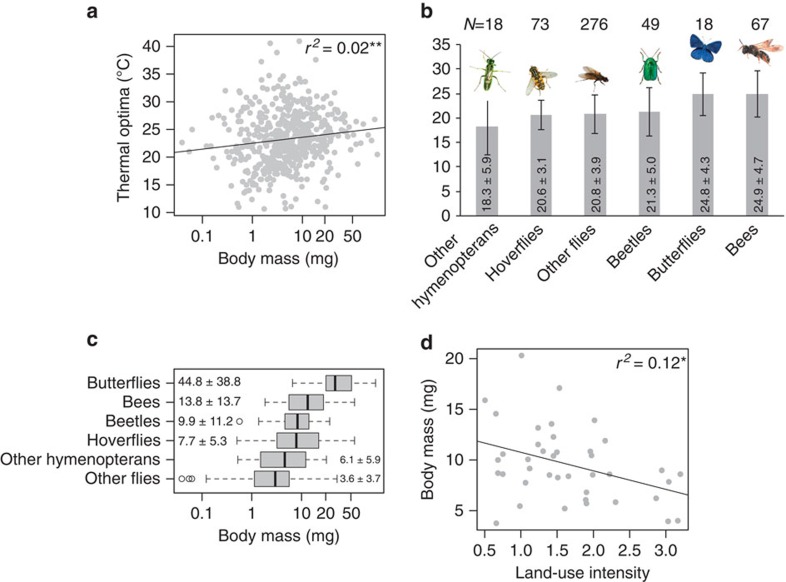
Role of body mass of pollinators for thermal niches. Effects of body mass (**a**) and taxa (**b**) on thermal optima. Body mass of different taxa (mean±s.d.) (**c**). Effects of land-use intensity on weighted mean body mass of the pollinators (**d**).

The species' body mass (log transformed) was a significant predictor of thermal optima: larger species preferred higher temperatures (lm: *F*_1,508_=3.0, *P*=0.002, Fig. 4a). Flies (excluding hoverflies), hymenopterans (excluding bees) and beetles were comparably small, whereas bees and butterflies were almost twofold larger (Fig. 4c). Pollinator communities in intensively used grasslands had a higher abundance of smaller species (lme, *F*_1,36_=9.3, *P*=0.004, Fig. 4d) while heavier species (butterflies and hoverflies) became less common. There were no changes of body mass with LUI within insect orders. Mean body mass differed between the two regions (lme, *F*_1,36_=15.0, *P*<0.001), but the effect of LUI was similar (interaction: *F*_1,36_=3.3, *P*=0.076). Changes in thermal optima and with increasing LUI were the same when we used measured body size instead of calculated body mass (see Supplementary Fig. 3).

There was no correlation between the total abundances of species and their thermal optima (Spearman rank, *r*_S_=0.04, *P*=0.40) and only a weak correlation between abundance and thermal niche breadth (*r*_S_=0.16, *P*<0.001). Land-use effects were independent of the local microclimatic conditions. The mean summer air temperature was not correlated with LUI (lm, *F*_1,32_=0.4, *P*=0.536), and there was only a non-significant trend that pollinators in warmer grasslands had higher thermal optima (*F*_1,32_=3.5, *P*=0.07). Hence, variation in thermal niches was independent of climatic conditions in the species habitats.

## Discussion

In theory, response diversity—variation in species-specific reaction norms of functionally redundant species—is important in stabilizing ecosystem functioning over time and against environmental variability^1^ (but see ref. 14). Based on thermal niches of a large number of pollinators, we modeled the thermal resilience of 40 local pollinator communities. We found that communities in intensively used grasslands had more variable temperature optima (hence, higher response diversity) as well as broader species-specific thermal niches (higher tolerance). Both contributed to a significant increase in projected thermal resilience with LUI—an effect mainly driven by flies.

Ambient temperature appears as the main predictor of the general activity of insect pollinators—as expected for ectotherms—when observations cover a broad daytime temperature range from 5 to 37 °C (see also ref. 31). The effect exceeds by far the influence of variation in flower cover or flower diversity; both are known to affect pollinator abundance in other studies^33^. In most pollinator studies temperature has not been taken into account, for example, as a covariate in comparisons of visitation rates or pollinator diversities. Restricting the observation of pollinators to a narrowly defined range of intermediate temperatures, however, may limit undesired variation. The strong dependence of ectotherms such as insects to ambient temperatures makes them potentially vulnerable to climatic changes. Besides an increase in average temperature, climate change models highlight an increasing variation of summer and winter temperatures^34^ and a higher frequency of extreme weather events. Given our findings, we might expect pollinator species at intermediate latitudes to be less susceptible to stress due to temperature variation, while tropical and desert species may not tolerate further warming if such warming exceeds critical temperature thresholds^18^,^35^. Generally, broader thermal niches and not only higher thermal optima may buffer species against climate change impacts^36^,^37^. Therefore, community resilience benefits from species with broad niches, but also from species covering very different niches (high complementarity). Although species with lower thermal optima became more common in grasslands with LUI, projected activities of pollinator communities during particularly warm temperatures (35–40 °C) were still not reduced compared with low-intensity grasslands. The increase in thermal niche breadth thus compensated for a directional shift towards colder thermal optima. Therefore, in these highly diverse communities in meadows and pastures, climate warming does not appear to restrict the activity of pollinators and their potential services.

Interestingly, variation in thermal niches in this study did not correspond to climatic differences across the grasslands (which were mostly relatively similar) nor between the regions. Instead, the composition of pollinator species was driven by other environmental filters related to LUI, and resulting effects on thermal community niches appeared as by-product of other traits such as body mass and taxonomic constraints.

Changes in composition of functional traits have been reported from different communities and contexts, usually defined for morphological or life-history traits^9^,^38^. High LUI has been found to reduce the diversity of species and their functional traits in several studies, a trend that has been termed ‘functional homogenization'^13^,^39^. Characteristic traits of communities may thus act as land-use indicators^40^. With increasing LUI, habitat or food specialists are often found to decline whereas generalists increase^41^. In parallel to the increase in generalized species (in terms of their thermal niche) in the grasslands investigated here, generalized butterflies (in terms of larval host plants) and other pollinators (in terms of flowers visited) became more common with increasing LUI, while specialists declined^31^,^42^. In arable land, insecticides additionally affect life-history traits of bees with negative consequences for pollination^43^.

Variation in thermal optima of pollinators was significantly related to body mass: lighter insects preferred lower temperatures. The increased surface / volume ratio of small and light animals could trigger higher water loss rates^44^, which corresponds to an avoidance of warmer temperatures. This rule might only apply to sufficiently warm conditions, as cold temperatures in higher altitudes or latitudes impose an energetic threshold, possibly restricting smaller bodied pollinators. Moreover, most flies in our study are relatively small compared with bees and butterflies. Colder community thermal optima in intensively used grasslands thus correspond to the dominance of small flies and the loss of pollinators that prefer warmer temperatures. The shift of body size with LUI towards a prevalence of small-sized species is a pattern that is already known from beetle assemblages^45^,^46^.

The abundance of butterflies and bees (in one region) decreased with the LUI, whereas flies compensated these losses by an increasing abundance. Thus total diversity remained unaffected. Differences in community niches thus did not reflect pure diversity effects rather than changes in species composition. The contrasting responses of declining plant and stable pollinator diversity confirmed earlier findings from the plots and other grasslands in the Biodiversity Exploratories^31^.

A special role comes to flies in our study as they are representing the largest share of the pollinator community particularly in intensively used grasslands. Especially in high altitude or latitude systems, or for example in island systems where other pollinators are rare, flies are known to be crucial for pollination^47^. But their role for other ecosystems is poorly investigated and thus probably largely underestimated. Flies are generally important pollinators, characterized by a high taxonomic diversity^48^ and high interaction frequencies^49^. They contribute in a major way to plant diversity as well as agricultural production. Not only occasionally studied syrphid flies, but also often more numerous non-syrphid flies contribute to pollination^50^. Flies are known to respond differently to environmental disturbance than for example bees^25^,^51^. Our results suggest that they also differ in thermal niches from other pollinators and, therefore, could maintain pollination services at low temperatures where bees or butterflies are inactive. Their dominance compared with other pollinators, but also shifts of species composition within the flies contributed to the higher thermal resilience of the communities. The same effects of LUI on thermal niches that we found for all pollinators together also apply to the flies alone, but not to the remaining taxa (although these were similarly variable in niche breadth and niche complementarity). This shows that the impact of land use on thermal niches is an effect within the heterogeneous flies rather than an effect of having more flies. Therefore flies could be highlighted as a main stabilizing factor of pollinator communities in managed grassland ecosystems. Flies are more generalized pollinators than most other taxa^31^ and may thus be less affected by declining plant species diversity. The diversity of hoverflies has declined much less (and partly even increased) in some regions over the last decades compared with the more specialized bees^25^. Open flowers such as *Asteraceae* became much more common in intensively used grasslands although total plant diversity strongly decreased with LUI in our study. They provide nectar and pollen that is easily accessible for short-tongued flies. In addition to adult diets, flies could additionally profit from increases in various larval resources and habitats^52^.

Differences in thermal niches between pollinator taxa are relevant for maintaining pollination across a range of weather conditions, which has been described in several case studies. For example, Vicens and Bosch^53^ showed that *Osmia cornuta* had greater weather tolerance than other species pollinating apple flowers. In almond orchards honeybees were replaced from wild pollinators at high wind speeds^10^. Weather also plays an important role in the pollination and yield of high bush blueberry, corresponding to variation in pollinator community composition^54^. Fründ *et al.*^16^ used between one and five pollinator species in experimental cages to study their pollination of several plants, and also fitted temperature activity curves for each pollinator species. The temperature range for which at least one species was active and could provide pollination services increased with the diversity of the community, which could contribute to a higher pollination success.

Other studies showed strong negative effects on pollinators or their pollination services as a response to habitat conversion or fragmentation^26^,^55^. The gradient in our study represents quantitative differences in grassland management intensity (fertilization, mowing, grazing), but did not involve unmanaged fallows or habitat conversion to arable land that are usually associated with strong biodiversity losses. Therefore, we caution against generalizations of our findings about resilient communities: in scenarios of stronger biodiversity losses and for taxa that suffer more substantially from land use, losses in stability are expected.

We conclude that an increase in thermal resilience of pollinators in intensively used grasslands is mainly driven by flies that profit from land use and compensate for losses of other taxa. The higher thermal resilience suggests that across the entire temperature range in the vegetation period, at least some species may continue to perform their functions. This highlights the relevance of response diversity for the resilience of ecosystems against variable conditions. The diversity of pollinators was high in all grasslands investigated here. However, in ecosystems with high losses of species and more severe human impacts, a more limited response diversity could put the maintenance of ecosystem functions at risk.

## Methods

### Study sites

Data were between May and September 2012 on grassland plots in the Hainich-Dün region in central Germany (within a radius of 35 km) and in the Schwäbische Alb in southwestern Germany (within a radius of 20 km, see maps in Supplementary Fig. 4). The plots are part of the Biodiversity Exploratories project^56^. In our study, 40 plots were selected along a land-use gradient from semi-natural to intensively managed grasslands. Land use can be characterized for each plot by a compound LUI index^57^ that integrates intensity of fertilization, mowing frequency and grazing intensity. For our analyses we use an averaged LUI from 2011 and 2012 to consider land-use management during the observation year and the previous year, as both may have a direct influence on pollinating insects with a predominantly annual life cycle. To test for potential long-term effects of land use, we repeated the analysis using a LUI that was averaged from 2006 (when plots were established) to 2012. General results were very similar (see Supplementary Table 4). To test whether effects are reproducible, we also repeated the analysis for an earlier pollinator survey in 2008 (ref. 31) from which the records from the Schwäbische Alb and Hainich were used. The majority of plots (49 out of 70) in this earlier dataset were represented by a single day (6 h) and thus only provide a seasonal snapshot of the community, but collecting methods and transects were the same^31^.

From the continuous records of climatic conditions by environmental monitoring units, we calculated the mean temperature for our focal plots from 1 May 2012 to 31 August 2012 at 10 cm above the ground, a zone with high temperature variation^58^. Mean air temperatures during these summer months ranged from 15.1 to 18.2 °C across the plots in the Hainich and from 16.6 to 18.2 °C in the Schwäbische Alb.

### Plant—pollinator interactions

We surveyed 149 plant-flower visitor interaction networks on 16 plots in the Alb and 24 plots in the Hainich. Size of grassland plots varied between 187.1 and 1.4 ha (mean: 28.4). We observed the plots repeatedly between one (four cases) and 13 times (median: 4 observation days per plot, corresponding to a total observation time mean of 24 h per plot) (see Supplementary Table 5). Each time a transect of about 300 m^2^ per plot was observed for 6 h between 08.00 and 14.00 (methods comparable with ref. 31). The transect was divided into 8 sectors of 25 m length and 3 m width. Each sector was observed for 15 min, three times a day. During these transects walks all flower visitors were collected. Only insects that touched reproductive parts of the flowers were considered. All animals that could not be identified visually in the field were sampled and identified to species level by taxonomists. All visitors in this analysis are known to pollinate flowers in general (but not necessarily all herbs in these grasslands). Non-pollinating taxa (for example, bugs) were excluded; Thysanoptera and pollen beetles (*Meligethes sp*.) were not counted; they are mostly hidden within flowers, hence their abundance and activity could not be reliably quantified across different plants.

To assess the importance of flower resources on the plots we counted floral units for all flowering plant species (excl. grasses) or estimated their number by extrapolation from a small area for highly abundant plants. Floral units were defined as one flower (for example, Asteraceae) or more flowers when flying would be necessary for pollinators to switch between flowers. For each plant species a characteristic flower was measured. In actinomorphic flowers, flowering area was calculated as a circle based on the flower diameter, whereas the flowering area of zygomorphic flowers was calculated as a rectangle based on flower length and width. Flower cover per species was calculated by multiplying the number of flowering units of a species by its flowering area. Results of each plant species were summed up to calculate the flower cover per plot (see Supplementary data 1). For reasons of time no new flower cover estimation was done when there were <4 days between the pollinator surveys.

### Pollinator species traits

Pollinator responses to temperature and body size as species traits were measured to investigate the role of different pollinators in managed grasslands. Air temperature was recorded at 15-min intervals at the height of the vegetation with the portable weather station TFA Nexus 35.1075 (TFA Dostmann GmbH & Co. KG, Germany). For each pollinator individual the actual temperature at which it was observed was assigned. Plots were repeatedly visited at different weather conditions, but even observations were not possible due to mowing and grazing. Across all sites we observed pollinators at air temperatures between 5 and 37 °C.

We measured body size of each one individual per species using a stereomicroscope with digital camera and software after calibration (Motic SMZ 168). Head, thorax and abdomen were measured separately before total body length was calculated as the sum, avoiding problems of variation in abdomen position and so. Body lengths for unavailable specimen were supplemented with values from the literature in 15 cases. All species names, body size and predicted body mass are listed in the Supplementary data 2. We converted body length (mm) of pollinator species to dry body mass (mg) using average body mass estimates of conversion equations for each insect order provided by Dillon and Frazier^59^. This method is recommended for community analyses^60^ since biomass is a better indicator of the functionality of a species within a community than its size.

### Thermal niche

Temperature-specific pollinator activity (*A*_T_) within intervals of 15 min was calculated by the sum of individuals per temperature *T* divided by the number of intervals in which this temperature was recorded. This standardization accounts for differences in the frequency at which temperatures were recorded. To characterize the thermal niche of each pollinator species we supplemented the data from 2012 by pollinator activities recorded together with air temperatures in an earlier study^31^ conducted in 2008, yielding activity data from a total of 35,875 pollinator individuals. The high number of individuals enabled us a detailed analysis of the temperature niches also of rare species. To characterize the pollinator community on a plot, all species and their number of individuals were pooled over the whole season in our target year 2012.

Most environmental niches of species are represented by unimodal functions, typically Gaussian curves of the activity or functional performance. Here the unimodal thermal niche of each species S is characterized by two parameters: the weighted mean μ_S_ temperature (*T*) at which it was recorded, and the weighted s.d. *σ*_S_ (Fig. 1). The mean μ_S_ represents the species' thermal optimum, *σ*_S_ its niche breadth. Therefore,


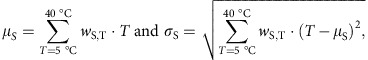


where *w*_S,T_ is the proportional weight (sum of weights=1 for each species S). Weighting is based on both the activity rate *A*_S,T_ (standardized number of individuals per 15 min per *T*) and the sample size *N*_S,T_ (number of individuals per *T*) as


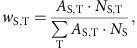


where *N*_S_ is the total number of individuals of the species S. This weighted approach considers the relative temperature preferences (rates) as well as the reliability (number of observations per temperature) to characterize a species' niche. To test the effects of individual weighting on the niche characterization, we also calculated μ_S_ with weights *w*_S,T_ based on activity rates *A*_S,T_ and on number of individuals *N*_S,T_ alone. Niche characterization was robust against the choices of weights. Mean μ_S_ based on these alternative weights are highly correlated to our preferred combined weighting (Spearman rank, *r*=0.96, *P*<0.001 for *A*_S,T_; *r*=0.92, *P*<0.001 for *N*_S,T_, *n*=511 species, respectively).

Each community (defined as a set of co-occurring species at a site) can be summarized by three parameters: the average species' optima, their variation across the species that defines their complementarity (that is, response diversity), and the mean niche breadths of the species. All measures are weighted by the proportional abundance of each species in the community *p*_S_. The weighted mean niche optimum (Fig. 1) across the species-specific μ_S_ in the community is defined as:


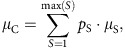


the weighted coefficient of variation (CV) in μ_S_ to represent the niche complementarity across species as


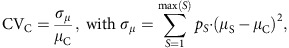


and the weighted mean niche breadth (*σ*_S_) as


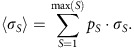


Generally, stabilizing effects of species diversity are suggested to be strongly influenced by abundance^61^. The extinction of a single abundant species can lead to a high impairment of ecosystem functioning if this species strongly contributes to the target process^62^. Therefore, we include species relative abundances (*p*_S_) in the analysis of thermal niches here. Weighting species by abundance also accounts for potential inaccuracies of thermal niches of species with few observations. Nevertheless, to examine a potential bias by rarely observed species, we repeated the linear mixed effect models after excluding 291 species with fewer than 5 individuals. Effects of LUI on thermal niches of pollinators remained largely unchanged for this reduced dataset, supporting the robustness of the weighted analysis (see Supplementary Table 6).

While weighting of the niche parameter by species abundances may better characterize communities, it does not consider possible compensatory dynamics. For instance, frequent species may become rare, and in turn rare species may become more frequent due to competitive release. Hence, we additionally calculated thermal niches for unweighted niche parameters and obtained similar results (see Supplementary Table 7).

As the community curve may be multimodal if variation in μ_S_ is large compared with *σ*_S_, the weighted s.d. of the community performance (*σ*_C_ in Fig. 1) does not fully characterize its resilience. Instead, the thermal resilience of the community *R*_C_ is defined as the integral of the summed species curves, hence


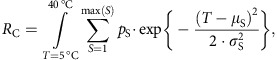


which is again weighted by *p*_S_, being the relative abundance of species *S*.

To facilitate comparisons of different communities that differ in the amplitude of the activity curves, *R*_C_ is standardized by dividing it by the maximum amplitude; this normalizes all communities to the same maximum of 1.

Note that this concept of thermal niches and resilience, as any trait dimension used in the context of response diversity so far, is based on fixed trait values per species—neglecting the potential of inter-population variability and individual plasticity^12^. The resulting estimations of resilience may thus be considered conservative, but this should not bias the relative differences between communities, that is, the main scope of our study. Conceptually, resilience can increase by two drivers: higher niche breadth and higher niche complementarity (Fig. 1). Niche complementarity and breadth are independent, since our definition of complementarity only focuses on variation in thermal optima rather than on niche overlap.

### Data analyses

Statistics were conducted in R 2.15.1 (ref. 63) We fitted a Gaussian function using the ‘nls' algorithm in R to describe the relationship between total pollinator activity and temperature. To estimate the goodness-of-fit, we derived an *r*^2^ from a Pearson's correlation between observed and fitted values. The effects of LUI on mean pollinator diversity and plant diversity per plot as well as the influence of flower cover on mean pollinator abundance per plot were assessed with linear models. We used the exponential form of Shannon diversity *e*^*H*'^ as measure for diversity to consider different abundances of species. To estimate the effects of LUI on the abundance of different pollinators, we divided the pollinators in different taxonomical groups (in analogy to Weiner *et al.*^31^) and analysed them separately with linear models: bees (67 species), beetles (49), butterflies (28), other flies (276), other hymenopterans (18) and hoverflies (73). Hoverflies and bees were separated from flies and hymenopterans, respectively, as they are commonly used as bioindicator taxa^25^. We transformed by arcsine square root the proportion of pollinator taxa in the community and the flower cover to meet the assumptions of homoscedasticity.

To estimate the main and interaction effects of LUI and region (Exploratory) and the main effect of species diversity on thermal optima, thermal niche breadth, thermal niche complementarity and community niche area, we fitted four lme. The mean air temperature during the observations on each plot was employed as random factor to control for a potential bias of conditions in each plot, but the general results remained unchanged when this random factor was removed. We ran analyses for all taxa, for flies (the dominant taxon) and all taxa excluding flies to identify whether flies are responsible for land-use effects.

## Additional information

**How to cite this article:** Kühsel, S. & Blüthgen, N. High diversity stabilizes the thermal resilience of pollinator communities in intensively managed grasslands. *Nat. Commun.* 6:7989 doi: 10.1038/ncomms8989 (2015).

## Supplementary Material

Supplementary Information Supplementary Figures 1-4 and Supplementary Tables 1-7

Supplementary Data 1 The diversity of flowering plant species for each plot

Supplementary Data 2 Species list of all pollinators and their body size

## Figures and Tables

**Table 1:** Determinants of thermal niches of pollinator communities.

**All taxa**					**Flies only**				
	**df**_**num**_	**df**_**den**_	***F***	***P*-value**		**df**_**num**_	**df**_**den**_	***F***	***P*-value**
*Thermal optima*					*Thermal optima*				
Taxa	5	168	16.4	**<0.001**	LUI	1	35	21.3	**<0.001**
LUI	1	36	6.8	**0.013**	Region	1	35	8.6	**0.006**
Region	1	36	2.8	0.105	LUI × Region	1	35	3.3	0.077
LUI × Region	1	36	0.2	0.638	Diversity	1	35	2.1	0.159
Diversity	1	168	0.5	0.486					
	
*Thermal niche breadth*					*Thermal niche breadth*				
Taxa	5	141	14.5	**<0.001**	LUI	1	35	3.7	0.063
LUI	1	36	11	**0.002**	Region	1	35	2.2	0.147
Region	1	36	1	0.315	LUI × Region	1	35	0.1	0.773
LUI × Region	1	36	0.2	0.64	Diversity	1	35	4.6	0.038
Diversity	1	141	0.8	0.388					
					
*Thermal niche complementarity*					*Thermal niche complementarity*				
Taxa	5	141	18	**<0.001**	LUI	1	35	11.1	**0.002**
LUI	1	36	15	**<0.001**	Region	1	35	0.7	0.416
Region	1	36	0.6	0.444	LUI × Region	1	35	0.2	0.687
LUI × Region	1	36	0.8	0.374	Diversity	1	35	2.2	0.146
Diversity	1	141	1.1	0.304					
					
*Community niche area*					*Community niche area*				
LUI	1	35	13.8	**<0.001**	LUI	1	35	4.7	**0.037**
Region	1	35	0.1	0.775	Region	1	35	2	0.171
LUI × Region	1	35	0.9	0.342	LUI × Region	1	35	0.8	0.227
Diversity	1	35	0.3	0.611	Diversity	1	35	1.5	0.366

LUI, land-use intensity.

Effects of pollinator taxa, LUI (average of 2011 and 2012), Exploratory (region) and pollinator diversity (*e*^*H*'^) on thermal responses of pollinators. Results are from linear mixed models. Bold values are statistically significant (*P*<0.05).

**Table 2:** Thermal niche of different pollinator taxa.

	**Niche breadth**		**Niche complementarity**	
	**mean**	**s.d.**	**mean**	**s.d.**
Butterflies	4.32	1.88	0.17	0.07
Hoverflies	4.48	2.06	0.18	0.07
Bees	4.50	1.85	0.18	0.07
Other flies	4.53	2.42	0.21	0.14
Other hymenopterans	4.56	1.73	0.23	0.12
Beetles	5.11	2.27	0.22	0.11

Mean and s.d. of niche breadth and niche complementarity for the six selected taxa.
