# Supplementary material for: High diversity stabilizes the thermal resilience of pollinator communities in intensively managed grasslands
Source: Nat Commun. 2015 Aug 10;6:7989. doi: 10.1038/ncomms8989 (PMC4918356; doi:10.1038/ncomms8989)
Supplement: Supplementary Information — Supplementary Figures 1-4 and Supplementary Tables 1-7 [file ncomms8989-s1.pdf]

Supplementary Figure 1| Changes in plant community with land-use intensity. The proportions of plant families of the total flower cover per plot and their changes with land-use intensity.

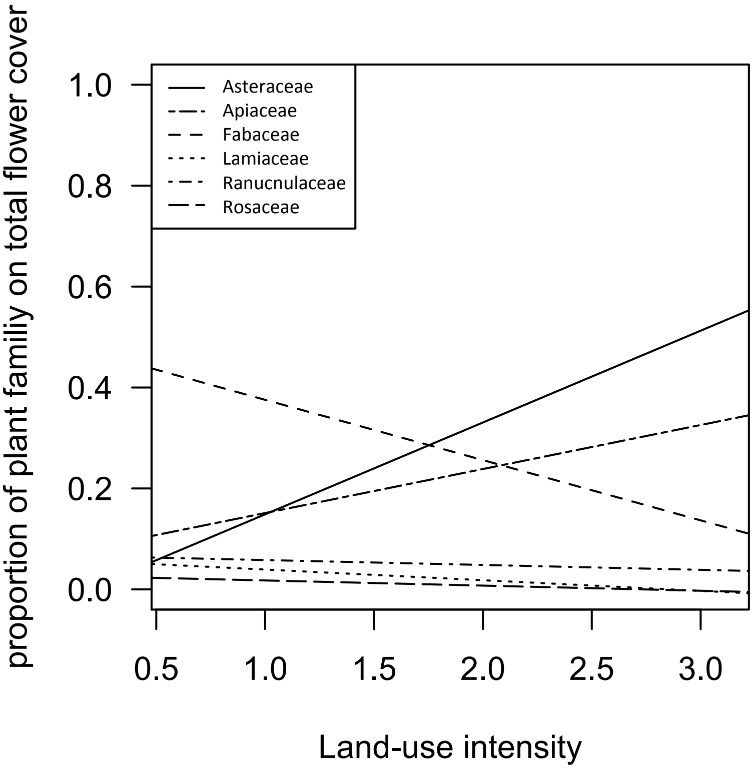

Supplementary Figure 2| Thermal niches of pollinator families. Variation in niche breadth and thermal niche complementarity of pollinator families within each of the four insect orders. Boxplots represent median, upper and lower quartiles and standard deviation of the species' values in each familiy.

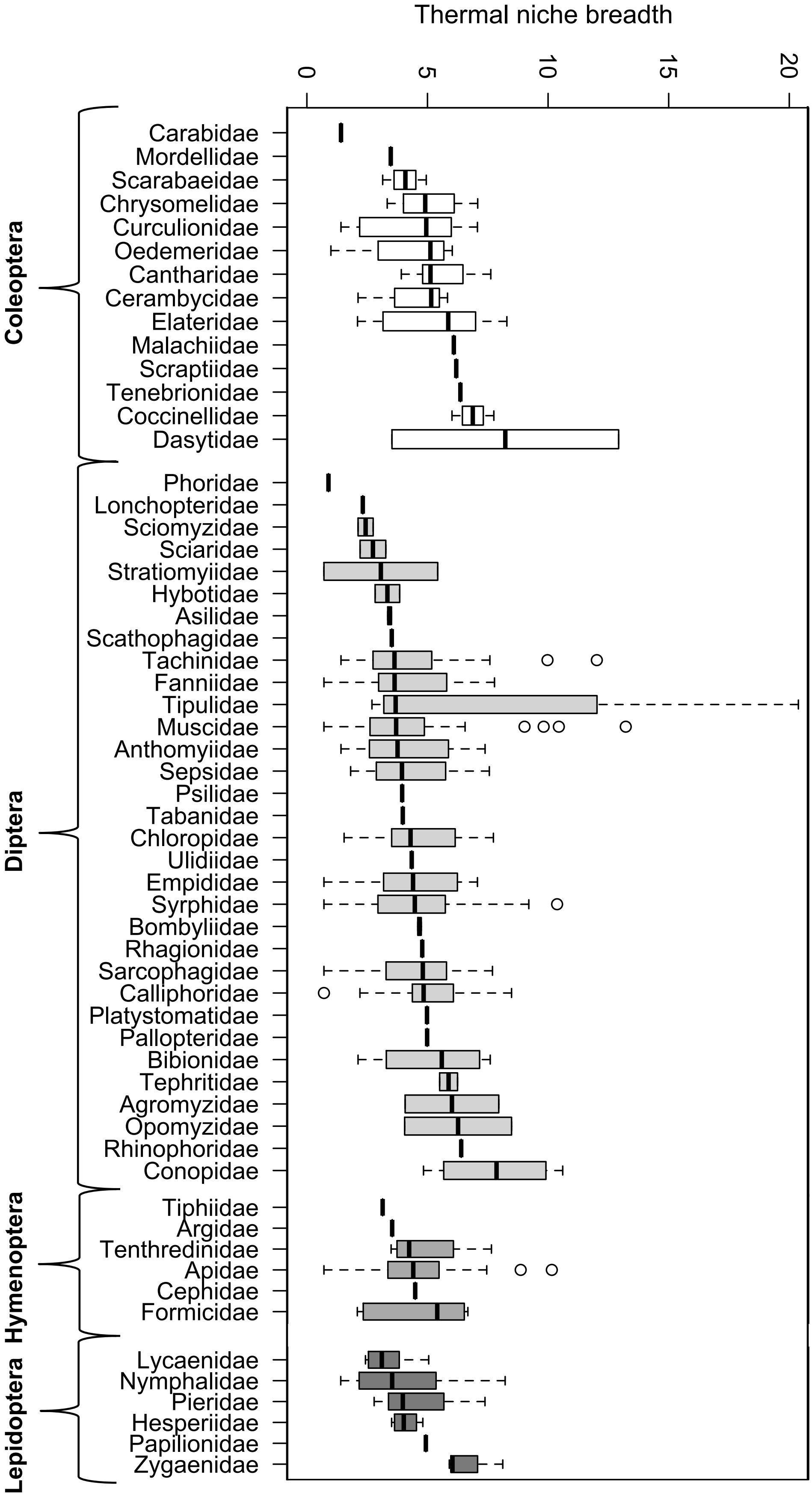

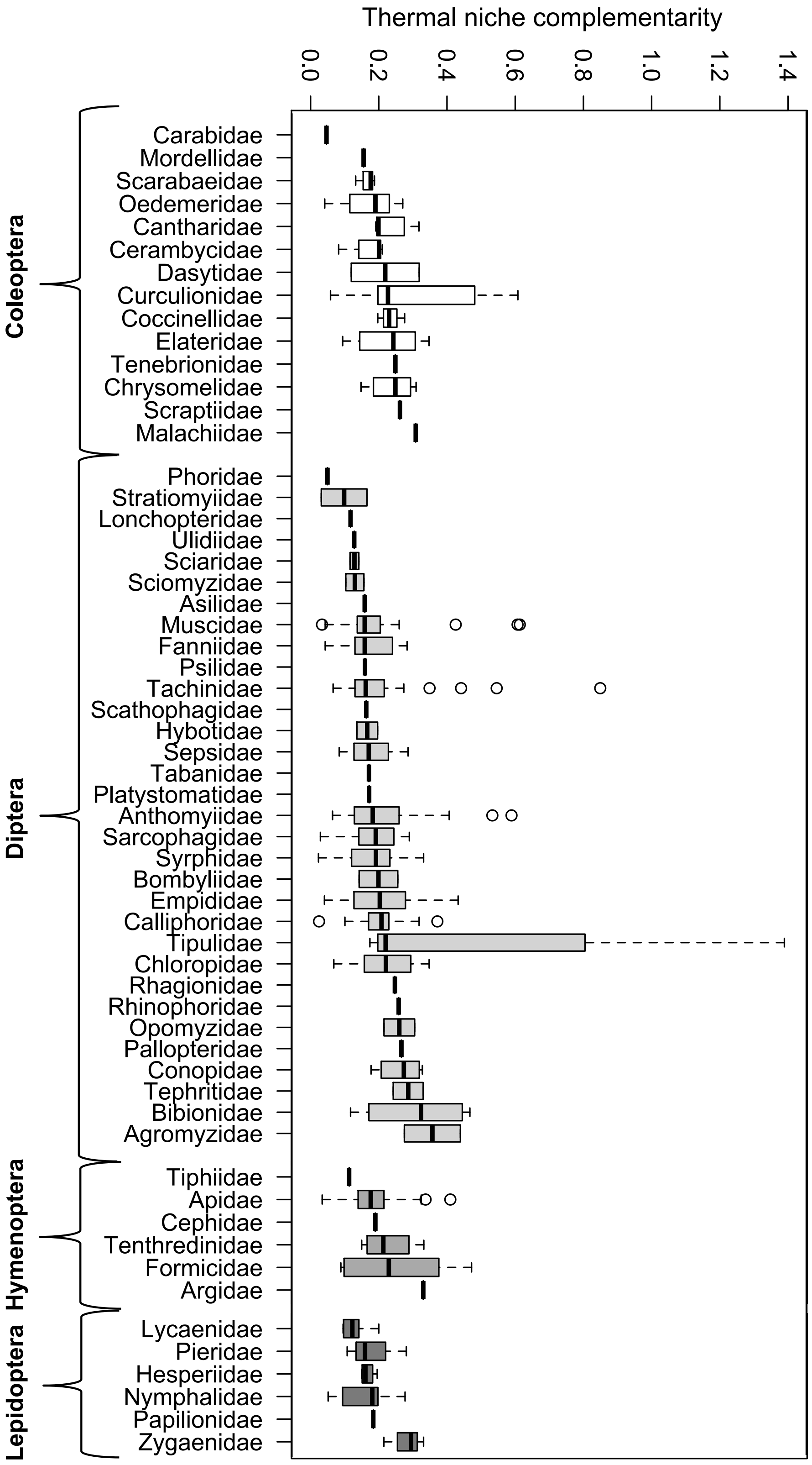

Thermal niche complementarity
0.0
0.2
0.4
0.6
0.8
1.0
1.2
1.4
Coleoptera
Carabidae
Mordellidae
Scarabaeidae
Oedemeridae
Cantharidae
Cerambycidae
Dasytidae
Curculionidae
Coccinellidae
Elateridae
Tenebrionidae
Chrysomelidae
Scraptiidae
Malachiidae
Diptera
Phoridae
Stratiomyiidae
Lonchopteridae
Ulidiidae
Sciaridae
Sciomyzidae
Asilidae
Muscidae
Fanniidae
Psilidae
Tachinidae
Scathophagidae
Hybotidae
Sepsidae
Tabanidae
Platystomatidae
Anthomyiidae
Sarcophagidae
Syrphidae
Bombyliidae
Empididae
Calliphoridae
Tipulidae
Chloropidae
Rhagionidae
Rhinophoridae
Opomyzidae
Pallopteridae
Conopidae
Tephritidae
Bibionidae
Agromyzidae
Hymenoptera
Tiphiidae
Apidae
Cephidae
Tenthredinidae
Formicidae
Argidae
Lepidoptera
Lycaenidae
Pieridae
Hesperiidae
Nymphalidae
Papilionidae
Zygaenidae

Supplementary Figure 3| Relationship between body size of pollinators, thermal niches and land-use intensity. (**a**) Effect of body size on thermal optima of species and (**b**) effects of land-use intensity on weighted mean body size across plots..

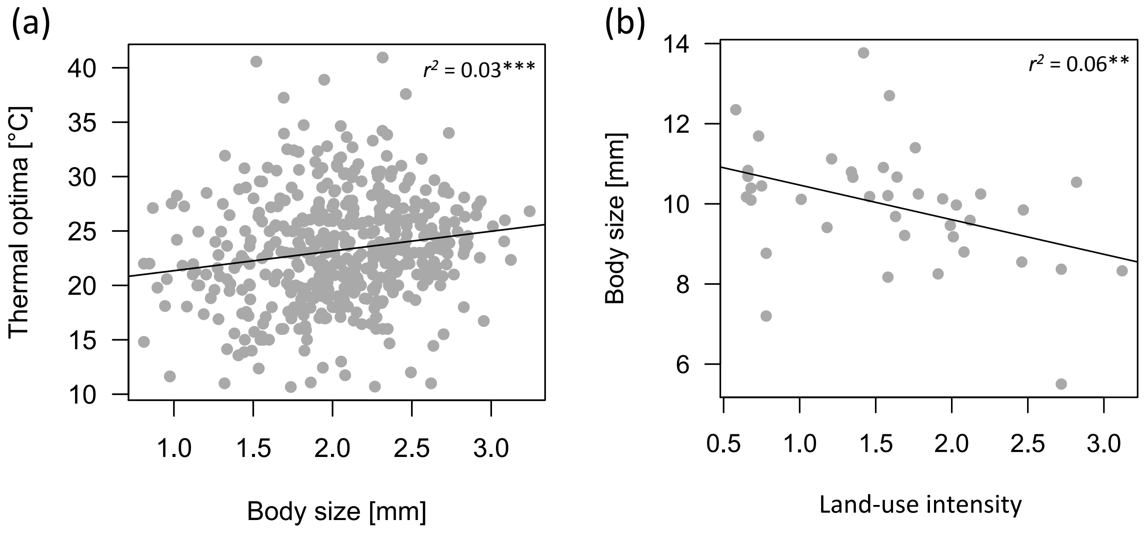

Supplementary Figure 4| Map of grassland plots. Location of the grassland plots used for pollinator sampling in Hainich (Thüringen, central Germany) and Swabian Alb (Baden-Württemberg, southwest Germany).

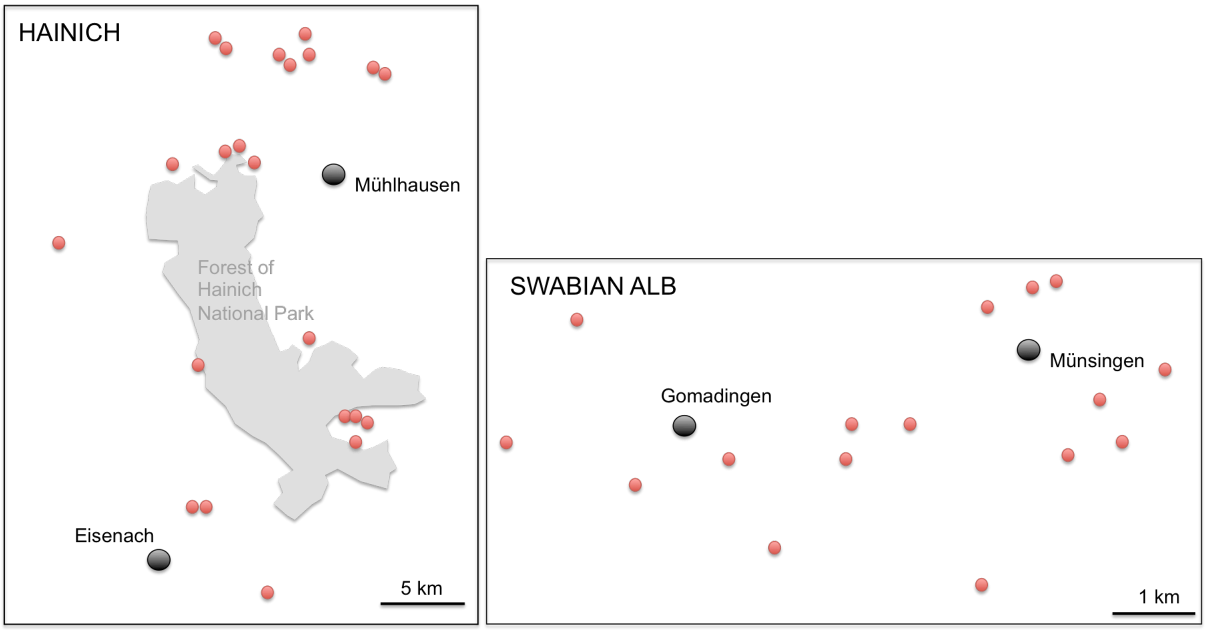

Supplementary Table 1| Compositional changes of plant communities with land-use intensity. Changes in the proportion of plant families of total flower cover per plot with increasing land-use intensity, assessed with linear models for each family. N = number of plots in which the family occurred. Predictors with $p < 0.05$ were considered to be significant (boldface).

	F	*p*	*N*
Asteraceae	13.3	**< 0.001**	35
Apiaceae	2.5	0.122	35
Fabaceae	4.3	**0.046**	37
Lamiaceae	3.9	0.055	34
Ranunculaceae	0.2	0.680	26
Rosaceae	2.2	0.151	23

Supplementary Table 2| Determinants of thermal niches of pollinator communities in 2008. Effects of land-use intensity (LUI), region and pollinator diversity ($e^{H'}$) on thermal responses of pollinators. Land use intensity was averaged over the years 2007 and 2008. Results are from linear mixed models. Predictors with $p < 0.05$ were considered to be significant (boldface).

	df_{num}	df_{den}	*F*	*p*
**Thermal optima**				
LUI	1	65	< 0.1	0.881
Region	1	65	< 0.1	0.849
LUI × Region	1	65	0.5	0.498
Diversity	1	65	< 0.1	0.922
**Thermal niche breadth**				
LUI	1	65	3.3	0.075
Region	1	65	1.4	0.248
LUI × Region	1	65	0.4	0.524
Diversity	1	65	7.1	**0.010**
**Thermal niche complementarity**				
LUI	1	65	3.1	0.083
Region	1	65	1.2	0.279
LUI × Region	1	65	0.6	0.452
Diversity	1	65	6.1	**0.016**
**Community niche area**				
LUI	1	65	1.8	0.185
Region	1	65	< 0.1	0.826
LUI × Region	1	65	1.2	0.279
Diversity	1	65	24.9	**< 0.001**

In 2008, 588 pollinator species were observed on 70 grassland plots in Hainich and Swabian Alb with the same method of pollinator sampling method like in this study. 47 of the 70 plots were just observed one day (6h) at different times of the year and different times of the day.

Supplementary Table 3| Determinants of thermal niches of pollinator communities without flies. Effects of land-use intensity (LUI), region and pollinator diversity ($e^{H'}$) on thermal responses of pollinators. Results are from linear mixed models. Flies were excluded from the analysis. Predictors with $p < 0.05$ were considered to be significant and bold print.

	df_{num}	df_{den}	*F*	*p*
**Thermal optima**				
LUI	1	35	7.9	**0.008**
Region	1	35	0.3	0.598
LUI × Region	1	35	0.3	0.580
Diversity	1	35	0.6	0.446
**Thermal niche breadth**				
LUI	1	34	2.2	0.144
Region	1	34	1.1	0.299
LUI × Region	1	34	0.2	0.658
Diversity	1	34	0.1	0.806
**Thermal niche complementarity**				
LUI	1	34	2.7	0.11
Region	1	34	1.1	0.313
LUI × Region	1	34	0.2	0.653
Diversity	1	34	0.1	0.790
**Community niche area**				
LUI	1	34	1.7	0.200
Region	1	34	0.5	0.501
LUI × Region	1	34	< 0.1	0.961
Diversity	1	34	< 0.1	0.731

Supplementary Table 4| Long term effects on thermal niches of pollinator communities. Effects of land-use intensity (LUI), region and pollinator diversity ($e^{H'}$) on thermal responses of pollinators. Land use intensity was averaged over the years 2006 to 2012. Results are from linear mixed models. Predictors with $p < 0.05$ were considered to be significant (boldface).

	df_{num}	df_{den}	F	p
**Thermal optima**				
LUI	1	35	12.1	**0.001**
Region	1	35	1.1	**0.002**
LUI × Region	1	35	10.3	**0.003**
Diversity	1	35	0.1	0.804
**Thermal niche breadth**				
LUI	1	35	12.7	**0.001**
Region	1	35	0.1	0.798
LUI × Region	1	35	1.2	0.282
Diversity	1	35	0.2	0.689
**Thermal niche complementarity**				
LUI	1	35	13.9	**< 0.001**
Region	1	35	0.3	0.567
LUI × Region	1	35	2.2	0.145
Diversity	1	35	0.2	0.67
**Community niche area**				
LUI	1	35	15.5	**< 0.001**
Region	1	35	< 0.1	0.853
LUI × Region	1	35	1.2	0.287
Diversity	1	35	0.4	0.530

Supplementary Table 5| Plots sampled. Number of surveys (6 h) per plot in Alb and Hainich.

Plot (Alb)	Number of surveys	Plot (Hainich)	Number of surveys
AEG01	4	HEG01	6
AEG02	4	HEG02	4
AEG03	10	HEG03	2
AEG04	4	HEG04	1
AEG05	10	HEG05	5
AEG06	3	HEG06	5
AEG07	11	HEG07	10
AEG08	7	HEG08	9
AEG09	13	HEG09	2
AEG16	4	HEG10	2
AEG17	6	HEG15	1
AEG22	1	HEG17	2
AEG26	6	HEG18	2
AEG27	2	HEG20	3
AEG29	4	HEG24	2
AEG48	8	HEG26	2
		HEG28	2
		HEG29	2
		HEG31	4
		HEG41	3
		HEG42	2
		HEG44	1
		HEG47	2
		HEG48	4

Supplementary Table 6| Determinants of thermal niches of a subset of pollinator communities. All pollinator species with less than 5 individuals were excluded from the analysis to avoid inaccurate thermal niches resulting from only few data points. Effects of land-use intensity (LUI), region and pollinator diversity ($e^{H'}$) on thermal responses of pollinators. Results are from linear mixed models. Flies were excluded from the analysis. Predictors with p < 0.05 were considered to be significant (boldface).

	df_{num}	df_{den}	F	p
**Thermal optima**				
LUI	1	36	11.3	**0.002**
Region	1	36	8.61	**0.006**
LUI × Region	1	36	16.71	**< 0.001**
**Thermal niche breadth**				
LUI	1	36	9.2	**0.005**
Region	1	36	0.1	0.761
LUI × Region	1	36	1.7	0.203
**Thermal niche complementarity**				
LUI	1	36	10.6	**0.002**
Region	1	36	< 0.1	0.96
LUI × Region	1	36	3.5	0.071
**Community niche area**				
LUI	1	36	4.1	**0.049**
Region	1	36	< 0.1	0.924
LUI × Region	1	36	< 0.1	0.851

Supplementary Table 7| Determinants of unweighted thermal niches of pollinator communities. Effects of land-use intensity (LUI), region and pollinator diversity ($e^{H'}$) on thermal responses of pollinators. Results are from linear mixed models. Thermal responses are not weighted by abundance of the individuals considering that frequency can change. Predictors with $p < 0.05$ were considered to be significant (boldface).

	dfnum	dfden	*F*	*p*
**Thermal optima (unweighted)**				
Taxa	5	168	14.8	**< 0.001**
LUI	1	36	8.7	**0.006**
Region	1	36	2.6	0.114
LUI × Region	1	36	< 0.1	0.953
Diversity	1	168	1.9	0.170
**Thermal niche breadth (unweigthed)**				
Taxa	5	141	16.6	**< 0.001**
LUI	1	36	10.3	**0.003**
Region	1	36	3.0	0.093
LUI × Region	1	36	0.1	0.740
Diversity	1	141	< 0.1	0.913
**Thermal niche complementarity (unweighted)**				
Taxa	5	141	19.8	**< 0.001**
LUI	1	36	16.8	**< 0.001**
Region	1	36	1.4	0.245
LUI × Region	1	36	0.1	0.768
Diversity	1	141	0.1	0.733
